# Regarding: “development and validation of MRI-based radiomics model for clinical symptom stratification of extrinsic adenomyosis”

**DOI:** 10.1080/07853890.2026.2617758

**Published:** 2026-02-01

**Authors:** Xin Feng, Huali Xiang, Yuan Yuan

**Affiliations:** ^a^ Department of Health Education, Jiangxi Maternal and Child Health Hospital, Nanchang, China; ^b^ Department of Gynecology, Jiangxi Maternal and Child Health Hospital, Nanchang, China; ^c^ Office of Medical and Nursing Administration, Jiangxi Maternal and Child Health Hospital, Nanchang, China

Dear Editor

We read with great interest the recent article by Sun et al. [1] entitled ‘Development and validation of MRI-based radiomics model for clinical symptom stratification of extrinsic adenomyosis’. This study represents an important advance towards precision gynaecology by integrating quantitative MRI radiomics with clinical parameters to classify symptom heterogeneity in extrinsic adenomyosis. We commend the authors for their innovative work bridging imaging analytics and clinical application.

First, while the study effectively demonstrates the potential of radiomics to distinguish among symptom subtypes – pain, abnormal uterine bleeding (AUB), infertility and asymptomatic cases – the reproducibility of extracted features may be limited by manual region-of-interest (ROI) delineation. As the authors acknowledged, manual segmentation is labour-intensive and subject to interobserver variability. We recommend that future studies adopt semi-automated or deep learning-based segmentation methods, which have proven effective in enhancing reproducibility and efficiency across multicenter MRI datasets [2].

Second, although the proposed clinical–radiomic nomogram achieved robust discriminatory performance, its single-centre retrospective design may introduce overfitting bias. Incorporating external validation cohorts, ideally through multicenter collaboration, would enhance model generalizability and clinical robustness. Additionally, performing calibration and decision curve analyses could further confirm the model’s practical utility in individualized patient management.

Third, the biological interpretability of radiomic features – particularly those derived from texture matrices such as GLCM and GLSZM – remains an important challenge. Correlating these imaging biomarkers with histopathological or molecular data, including hormone receptor profiles and extracellular matrix remodelling indicators, could clarify the mechanisms underlying symptom diversity. Visualizing feature importance or applying heatmap-based model interpretation could also increase clinician confidence and facilitate clinical implementation.

Finally, this study holds substantial translational promise. A validated, noninvasive MRI-based stratification tool could refine preoperative counselling, guide individualized medical or surgical strategies, and reduce unnecessary interventions. Future research should aim to integrate this model within standardized frameworks, such as the evolving FIGO classification of adenomyosis, to promote cross-institutional comparability and clinical adoption.

In summary, Sun et al. have made a valuable contribution to understanding symptom heterogeneity in adenomyosis through advanced radiomics modelling. Further optimization *via* automated segmentation, multicenter validation and biological correlation will accelerate the translation of this technology from research to clinical practice.

## Data Availability

Data sharing does not apply to this article, as no data were created or analyzed in this study.
